# The European Public Health Community is mourning Richard Horst Noack

**DOI:** 10.1093/eurpub/ckad099

**Published:** 2023-06-28

**Authors:** Thomas E Dorner, Dineke Zeegers Paget

**Affiliations:** Austrian Public Health Association, Vienna, Austria; European Public Health Association, Utrecht, The Netherlands; European Public Health Association, Utrecht, The Netherlands

On May 19, 2023, EUPHA’s past president and ÖGPH’s honorary president—Professor Richard Horst Noack—passed away in his hometown Graz, Austria.

Horst Noack joined the EUPHA Executive Council in 2004 as president-elect and was EUPHA president in 2006. In 2005, Horst was the president of the EUPHA Conference, organized in Graz, Austria. The main theme of the 2005 conference ‘Promoting the Public’s Health: reorienting health policies, linking health promotion and health care’ reflected his cross-sectional vision. Under his leadership, the 2005 Graz conference had some great innovations. It was the first time that a EUPHA conference was organized by three countries (Austria, Croatia and Slovenia) and it was also the first time that one conference track was dedicated to the European Commission’s efforts to strengthen the field of public health, providing a platform for intersectorial discussion and, first-hand information on five European public health programmes and funding procedures. It was also the first time that a big public health event was organized in Austria. Horst remained active for EUPHA giving advice for years and even being on the Local Support Committee of the EPH conference 2016 in Vienna.

Horst Noack grew up in a rural region in Sachsen in East Germany. His parents were craftsmen and small-scale farmers. Together with two fellow students at the Technical University of Dresden he fled 19-year-old in 1957 from the German Democratic Republic (GDR) on a bicycle only with his work clothes to West Berlin, the suitcase with clothes and most important documents were already ‘drüben’ (over there).

As an officially recognized GDR refugee Horst would have been allowed to complete his Engineering studies in the Federal Republic of Germany, but he chose to study Medicine instead at Universities in Bonn and Tübingen. Horst also studied Psychology and Methods of Social Science Research at the Iowa State University in Ames in the USA. After completing his studies, he set up and headed the Department of Health Research at the Institute for Social and Preventive Medicine at the University of Bern in Switzerland. From 1992, Noack was appointed as a Professor of Social Medicine and Epidemiology in Graz, Austria, where he retired in 2005. He has also held various positions at the World Health Organization.

Horst Noack played a pioneering role in the development of public health, both nationally and internationally. He consolidated the term ‘New Public Health’ in order to further develop public health away from a medically patriarchal eminence-based perspective towards a modern interdisciplinary scientific discipline.

Public health in Austria is inextricably linked to his person and his name. He was a co-founder of the Austrian Public Health Association (ÖGPH). He was the first ÖGPH president and, as honorary president until his death, still had sympathetic ties to ÖGPH. His most important goal in the founding years was the development of part-time master’s course in public health based on the Swiss model, which was finally established in Graz for the first time in Austria. Later, he also co-founded the education program Public Health Governance in Vorarlberg.

His commitment to the training of a scientifically active public health work force, the creation of structured foundations for health promotion—he was significantly involved in the development of the Ottawa Charter of the WHO in 1986—and his numerous publications and book contributions make him one of the most important personalities in the international Public Health. To enumerate all his activities and merits would require writing a book. His guiding principle, however, that years of life gained should also be years of healthy life, has become a basic principle of health promotion and prevention.

Anyone who knew him and, above all, anyone who was fortunate enough to work with him will remember his charismatic, clever, educated and lovable personality. We will miss him greatly.

