# Correction to: The European Public Health Community is mourning Richard Horst Noack

**DOI:** 10.1093/eurpub/ckad165

**Published:** 2023-09-15

This is a correction to: Thomas E Dorner, Dineke Zeegers Paget, The European Public Health Community is mourning Richard Horst Noack, *European Journal of Public Health*, Volume 33, Issue 4, August 2023, Page 749, https://doi.org/10.1093/eurpub/ckad099

In the originally published version of this Editorial, the second author’s surname was spelled incorrectly as Padget. The author’s correct name is Dineke Zeegers Paget.

This error has been corrected online.

